# Utilization of Waste Bamboo Fibers in Thermoplastic Composites: Influence of the Chemical Composition and Thermal Decomposition Behavior

**DOI:** 10.3390/polym12030636

**Published:** 2020-03-11

**Authors:** Chin-Hao Yeh, Teng-Chun Yang

**Affiliations:** Department of Forestry, National Chung Hsing University, Taichung 402, Taiwan; harrison19960219@gmail.com

**Keywords:** bamboo-plastic composites (BPCs), waste bamboo fibers, chemical composition, physico-mechanical properties, thermal decomposition kinetics

## Abstract

In this study, four types of waste bamboo fibers (BFs), Makino bamboo (*Phyllostachys makinoi*), Moso bamboo (*Phyllostachys pubescens*), Ma bamboo (*Dendrocalamus latiflorus*), and Thorny bamboo (*Bambusa stenostachya*), were used as reinforcements and incorporated into polypropylene (PP) to manufacture bamboo–PP composites (BPCs). To investigate the effects of the fibers from these bamboo species on the properties of the BPCs, their chemical compositions were evaluated, and their thermal decomposition kinetics were analyzed by the Flynn–Wall–Ozawa (FWO) method and the Criado method. Thermogravimetric results indicated that the Makino BF was the most thermally stable since it showed the highest activation energy at various conversion rates that were calculated by the FWO method. Furthermore, using the Criado method, the thermal decomposition mechanisms of the BFs were revealed by diffusion when the conversion rates (α) were below 0.5. When the α values were above 0.5, their decomposition mechanisms trended to the random nucleation mechanism. Additionally, the results showed that the BPC with Thorny BFs exhibited the highest moisture content and water absorption rate due to this BF having high hemicellulose content, while the BPC with Makino BFs had high crystallinity and high lignin content, which gave the resulting BPC better tensile properties.

## 1. Introduction

Owing to the depletion of fossil fuels and the growth of environmental awareness, the effective utilization of forestry waste residues is a notable issue. In Taiwan, the waste residues that are produced from bamboo or woody processing are buried, incinerated, or burned in boilers [1]. According to the literature [2], this waste can be recycled and reused to contribute more economic and social benefits. Bamboo is a renewable material and grows quickly compared to other plants. Additionally, bamboo possesses approximately 60% cellulose with high lignin content and a longitudinal alignment of fibers, which includes a relatively small microfibrillar angle [3,4,5], resulting in highly specific mechanical properties. In Taiwan, Makino bamboo (*Phyllostachys makinoi*), Moso bamboo (*Phyllostachys pubescens*), Ma bamboo (*Dendrocalamus latiflorus*), and Thorny bamboo (*Bambusa stenostachya*) are common economical and popular bamboo species. Among these bamboo species, Moso bamboo is the most globally harvested bamboo. Therefore, several studies have investigated the chemical, anatomical, physical, and mechanical properties of Moso bamboo [4,5,6,7]. In Taiwan, production from Makino bamboo has accounted for more than 80% of gross bamboo production in the past decade [8]. Chung and Wang [6] reported that the flexural properties of Makino bamboo were greater than those of Moso bamboo since the chemical composition of Makino bamboo has higher holocellulose and α-cellulose contents. To effectively utilize waste bamboo residues, polymer composites composed of natural fibers are of significant interest and have been identified as emerging trends in composite science. Furthermore, the addition of natural fibers leads the composite to being an eco-friendly material and reduces the cost of the final composite products due to the numerous advantages of the natural fiber, including low density, high toughness, good specific strength properties, biodegradability, and renewability [9,10,11,12,13]. These composites are widely applied in residential markets and construction industries as window framing, decking, and fencing. Among several natural fibers, bamboo fiber (BF) reinforcement has significant potential for improving the properties of polymer composites due to its excellent characteristics [14,15,16,17]. Previous studies [18,19,20] indicated that the chemical composition and morphology of the fiber, fiber-matrix stress transfer efficiency, and microstructure and void content of the composite are factors that significantly affect the physical and mechanical properties of wood-plastic composites (WPCs). Similarly, the chemical components, including cellulose, hemicellulose, lignin, and extractives, of different bamboo fibers could result in distinct differences in the performance of bamboo–polypropylene composites (BPCs). Moreover, thermal degradation of natural fibers occurs during the manufacturing process of a composite [21,22]. Hence, the thermal decomposition mechanisms from kinetic analyses are crucial for providing information on the thermal degradation processes of fibers. Furthermore, the kinetic modeling of decomposition could help the design of composite processes and is useful for further understanding the thermal stability of the composite. The thermal decomposition kinetics can be evaluated by the isoconversional method, which includes model-free kinetics for determining the activation parameters [23,24]. Some studies have indicated that the thermal decomposition kinetics are influenced by the properties of the fibers such as chemical composition, moisture content, density, and crystallinity [25,26,27]. Criado et al. [28] proposed several kinetic equations to explain the thermal decomposition mechanisms of solid-state reactions, such as diffusion, nucleation and growth, random nucleation, and phase boundary control. To the best of our knowledge, there is little information available on the properties of BPCs with various waste BFs obtained from these four bamboo species in Taiwan. Accordingly, the aim of the present study was to focus on the effects of the chemical composition and thermal decomposition behavior of different BFs on the physical and mechanical properties of BPCs. Additionally, the thermal stability and kinetic mechanism of the BF were determined using thermogravimetric (TG) analysis by the isoconversional method.

## 2. Materials and Methods

### 2.1. Materials

Waste bamboo shavings from various 3-year-old Makino bamboo (*Phyllostachys makinoi*), Moso bamboo (*Phyllostachys pubescens*), Ma bamboo (*Dendrocalamus latiflorus*), and Thorny bamboo (*Bambusa stenostachya*) culms were provided by a local bamboo-processing factory (Nantou County, Taiwan). The BFs were prepared by hammer-milling and sieving between 6 and 16 mesh (*ϕ*1.00–3.35 mm). The polypropylene (PP) used in this study was purchased from Yung Chia Chemical Industries Co., Ltd. (Taipei, Taiwan). The density, melting temperature, and melt flow index of the PP were 915 kg/m^3^, 145 °C, and 4–8 g/10 min, respectively. The PP pellets were ground in an attrition mill to reduce their size to between 20 and 80 mesh (*ϕ*180–850 μm). The solvents (methanol and toluene) and chemicals (glacial acetic acid, sodium chlorite, and sulfuric acid) were purchased from Sigma-Aldrich Chemical Co. (St. Louis, MO, USA).

### 2.2. Manufacturing Process of the Bamboo–Polypropylene Composites (BPCs)

The weight ratio of oven-dried BF (moisture content < 3%) to PP was 50/50 for manufacturing the BPCs through the flat-platen pressing process, designated BPC_Makino_, BPC_Moso_, BPC_Ma_, and BPC_Thorny_. All the BPCs were produced in a two-step pressing process as follows: (1) hot pressing (2.9 MPa) at 180 °C for 3 min and (2) finishing by cold pressing until the temperature decreased to 50 °C. The expected density of the BPCs was 0.8 g/cm^3^. The expected dimensions of the BPCs were 300 mm × 200 mm with a thickness of 3 mm.

### 2.3. Chemical Composition Analysis

According to ASTM D1107-96, ASTM D1104-56, and ASTM D1106-96, the contents of extractives, holocellulose, and Klason lignin were determined for the various BFs. The chemical composition contents were expressed as a percentage of the initial oven-dried weight.

### 2.4. X-ray Diffraction (XRD)

X-ray diffractograms (XRD) were collected with an MAC science MXP18 instrument (Tokyo, Japan) using Ni-filtered CuK_α1_ radiation (λ = 0.1542 nm) at 40 kV and 30 mA. The intensities of the XRD patterns were recorded in the 2θ range of 4–40° with a scan rate of 2°/min. The crystallinity index (CrI) of the BF was calculated according to the following equation [29]:

CrI (%) = 100 × (*I*_200_ − *I*_am_)/*I*_200_ (1)

where *I*_am_ is the intensity of diffraction of the amorphous material at 2θ = 18.3°, and *I*_200_ is the intensity of the 200 lattice reflection of the cellulose crystallographic form at 2θ = 22°.

### 2.5. Thermal Decomposition Kinetics Analysis

A Perkin Elmer Pyris 1 instrument (Shelton, CT, USA) was used to investigate the thermal properties of various BFs. A total of 3 mg of BF was heated in a nitrogen atmosphere (20 mL/min) from 50 to 600 °C at various constant heating rates of 5, 10, 20, 30, and 40 °C/min. The data obtained from the TG curves were used to calculate the kinetic parameters. The conversion rate (*α*) can be defined as:

(2) $$\alpha =\frac{m_{0}-m_{t}}{m_{0}-m_{f}}$$

where *m*_0_ is the initial weight of the sample, *m_f_* is the final residual weight, and *m*_t_ is the weight of the pyrolyzed sample at time *t*. The fundamental equation for a dynamic TG analysis in a nonisothermal experiment can be generally written as follows:

(3) $$\frac{d\alpha }{dT}=\frac{A}{\beta }\exp\left(-\frac{E_{a}}{RT}\right)f\left(\alpha \right)$$

where *T* is the absolute temperature (K), *A* is the pre-exponential factor (min^−1^), *β* is the heating rate (= d*T*/d*t*), *E*_a_ is the activation energy (kJ/mol), *R* is the gas content (8.314 J/ K/mol), and *f*(*α*) is the reaction model. Additionally, the integrated form of Equation (3) with a constant heating rate can be expressed in Equation (4):

(4) $$g\left(\alpha \right)=\int_{0}^{\alpha }\frac{d\alpha }{f\left(\alpha \right)}=\int_{0}^{T}\frac{A}{\beta }\exp\left(-\frac{E_{a}}{RT}\right)dT=\frac{E_{a}}{\beta R}\frac{\exp\left(-x\right)}{x}\pi \left(x\right)$$

where *x* = *E*_a_/*RT* and *π*(*x*) is the rational approximation of Senum and Yang [30]. According to Equation (4), the isoconversional Flynn–Wall–Ozawa (FWO) method can be transformed to estimate the activation energy (*E*_a_) value for the thermal decomposition process of the BF. This method is represented by the following equation [31,32]:

(5) $$\log\beta =\log\left(\frac{AE_{a}}{g\left(\alpha \right)R}\right)-2.315-0.4567\frac{E_{a}}{RT}$$

For various heating rates (*β*) and a given conversion rate (*α*), a linear relationship is observed by plotting log *β* versus 1/*T*, and the *E*_a_ value is calculated from the slope of the straight line [31,32,33,34,35]. The reaction mechanism of the decomposition process is determined by the Criado method [28,36], which assumes that the *Z_m_*(*α*) master curves are a convolution of the functions *f*(*α*) and *g*(*α*) corresponding to the different models listed in Table 1 [37,38]:

*Z_m_*(*α*) = *f*(*α*)*g*(*α*) (6)

On the other hand, the experimental *Z_e_*(*α*(*T*)) function can be obtained from Equation (6) by combining Equations (3) and (4):

(7) $$Z_{e}\left(\alpha \left(T\right)\right)=\left[\frac{d\alpha }{dT}\exp\left(\frac{E_{a}}{RT}\right)\right]\left[\frac{E_{a}}{R}\frac{\exp\left(-x\right)}{x}\pi \left(x\right)\right]$$

In this study, the fourth degree rational expression of Senum and Yang [30] was used, in which the percentage deviation is less than 10^−5^% when *x* > 20. This *π*(*x*) is expressed as follows:

(8) $$\pi \left(x\right)=\frac{x^{3}+18x^{2}+86x+96}{x^{4}+20x^{3}+120x^{2}+240x+120}$$

### 2.6. Determination of BPC Properties

The density, moisture content (MS), and water absorption rate (WAR) were determined according to ASTM D2395-17 and ASTM D1037-12. The ASTM D 638-14 and ASTM D 790-17 methods were implemented using a universal testing machine (Shimadzu AG-10kNX, Tokyo, Japan) to determine the tensile properties and flexural properties of the BPCs, respectively. The tensile properties, including the tensile strength (TS) and tensile modulus (TM), were assessed with type I dumbbell-shaped samples at a loading speed of 5 mm/min and a gage length of 57 mm. The modulus of rupture (MOR) and modulus of elasticity (MOE) were obtained using a three-point static flexural test with dimensions of 80 mm × 10 mm × 3 mm at a loading speed of 1.28 mm/min and a span of 48 mm. All the samples were conditioned at 20 °C and 65% relative humidity (RH) for two weeks prior to testing.

### 2.7. Analysis of Variance

All of the results are expressed in terms of the mean ± the standard deviation (SD). The significance of the differences was calculated using Scheffe’s test; *p* < 0.05 was considered to be significant.

## 3. Results and Discussion

### 3.1. Chemical Composition and Thermal Stability of Various BFs

**Figure 1:**
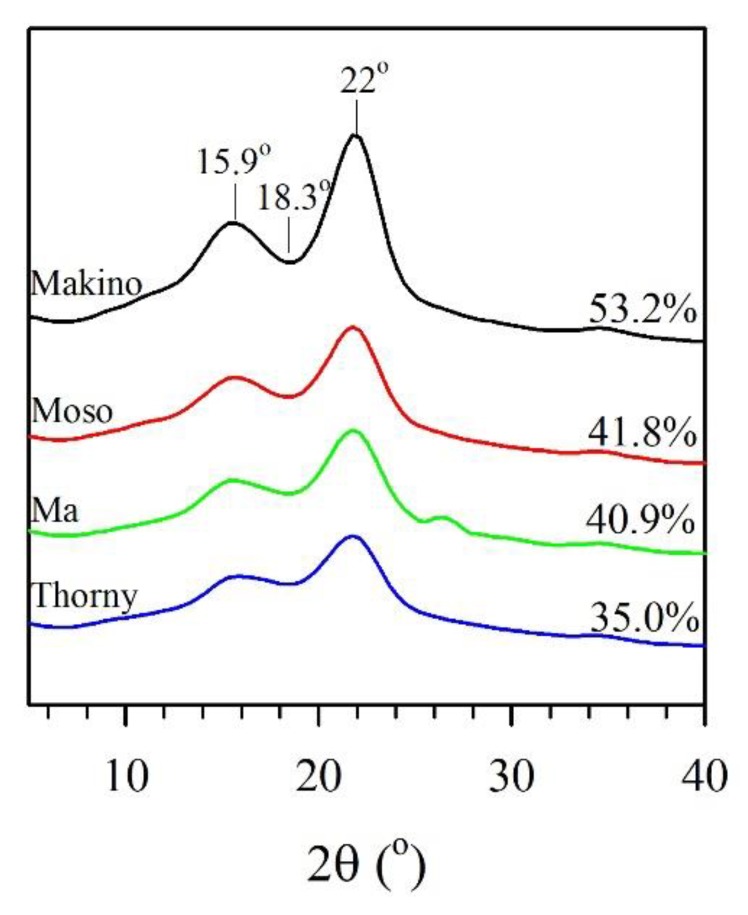
**X-ray diffraction** (XRD) patterns and crystallinity indexes (CrIs) of various BFs.

The chemical compositions of various BFs are listed in Table 2. The extractive contents of the Makino, Moso, Ma, and Thorny BFs were 2.9, 3.8, 8.5, and 6.9%, respectively, which yields an order of Makino < Moso < Thorny < Ma. It is noted that their holocellulose contents were in the order of Makino > Ma > Moso > Thorny (the values were 62.5, 58.0, 57.1, and 56.0%, respectively). This result indicated that the Makino BF had the highest holoceullose content among all of the samples. Additionally, it is worth noting that the Moso BF revealed the lowest lignin content (24.5%), while there were no signifcant differences among the lignin contents of Makino, Ma, and Thorny BFs, which were in the range of 28.1% to 30.7%. As shown in Figure 1, the crystallinity indexes (CrIs) of various BFs were calculated by the XRD patterns. The major peaks of cellulose crystal diffraction were observed for all the samples at approximately 15.9° (101/10$\overset{¯}{1}$ lattice planes) and 22° (200 lattice plane), whereas the diffraction value at 18.3° represented the amorphous region [8]. According to Equation (1) described above, the CrI values were 53.2%, 41.8%, 40.9%, and 35.0% for Makino, Moso, Ma, and Thorny BFs, respectively. A parameter termed the CrI has been used to describe the relative amount of crystalline material in cellulose. Therefore, this result illustrated that the Makino BF had the highest ordered cellulose content among all the samples, while the lowest amount of ordered cellulose appeared for the Thorny BF.

**Figure 2:**
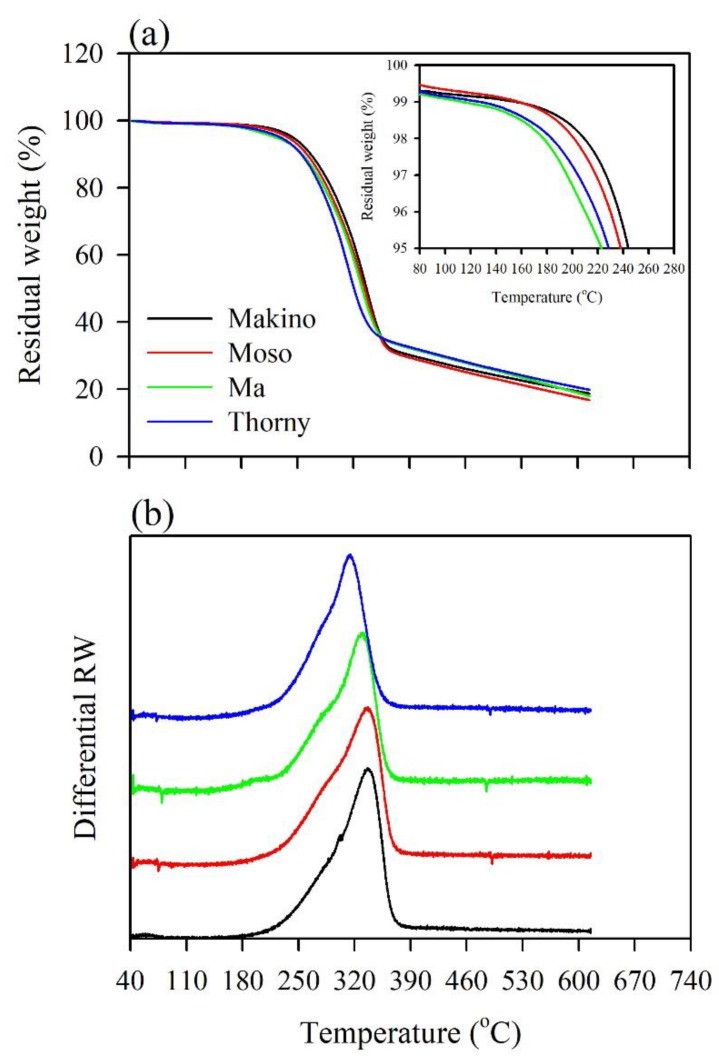
Thermogravimetric (TG) curves of various BFs at a heating rate of 5 °C/min. (**a**) Residual weight (RW) curves; (**b**) differential RW curves.

In this study, TG analysis was used at various thermal decomposition temperatures to interpret the preliminary evaluation of the thermal stabilities of the BFs and their BPCs during composite processing. Figure 2 shows the residual weight (RW) and differential RW curves of the different BFs from the results of the TG analysis. Órfão et al. [26] and Yang et al. [39] revealed that the thermal decomposition of wood is separated into three stages in the differential RW curve. The first stage corresponds to water evaporation, with a temperature range of 60–120 °C. The second stage simultaneously includes the total decomposition of hemicellulose and cellulose and the partial decomposition of lignin, at temperatures from 210–370 °C. During the third stage in the range of 370–480 °C, the remaining lignin decomposition and the combustion of the residues occur. As shown in detail in Figure 2a, the RW curves showed that various BFs started to exhibit significant weight loss at approximately 140–180 °C. Additionally, the temperatures at which the sample lost 3% (*T*_3%_) of its weight were 226, 219, 196, and 204 °C for the Makino, Moso, Ma, and Thorny BFs, respectively. The results demonstrated that the Ma BF exhibited the lowest *T*_3%_ value among all the samples. This result is related to the Ma BF having the highest extractive content, as shown in Table 2. It is well-known that extractive decomposition occurs at lower temperatures since extractives are compounds with lower molecular weights than the other chemical components. Previous studies [40,41,42] indicated that extractives could promote the ignitability of wood at lower temperatures and accelerate wood degradation due to their higher volatility. Furthermore, the Makino BF had the highest *T*_3%_ value (226 °C) and thus had higher thermal stability relative to the other BFs. This result is associated with the Makino BF having the lowest content of extractives. Figure 2b presents the differential RW curves of various BFs. These curves displayed a slight shoulder in the range of 250–300 °C, and remarkable peaks were observed in the range of 310–340 °C. According to the pyrolysis behaviors of the three main components (cellulose, hemicellulose, and lignin) in the differential RW curves, hemicellulose and cellulose were decomposed in active pyrolysis in the ranges of 220–315 °C and 315–400 °C, respectively [39]. The thermal decomposition of lignin occurs in active and passive pyrolysis over a wide temperature range from 160–900 °C without characteristic peaks [39]. As shown in Figure 2b, the hemicellulose decomposition in the Thorny BFs occurred at relatively low temperatures (215–285 °C), and the temperature of its characteristic peak (315 °C) was lower than those of the other BFs. This behavior may be related to the higher content of hemicellulose in Thorny BFs relative to those in the other BFs. Hence, this result indicates that a higher content of hemicellulose causes cellulose decomposition at a lower temperature and accelerates the thermal degradation of a BF. John and Thomas [43] explained that hemicellulose comprises a random amorphous structure and is more easily degraded than the other components at its thermal decomposition temperatures (220–315 °C).

### 3.2. Thermal Decomposition Kinetics of Various BFs

**Figure 3:**
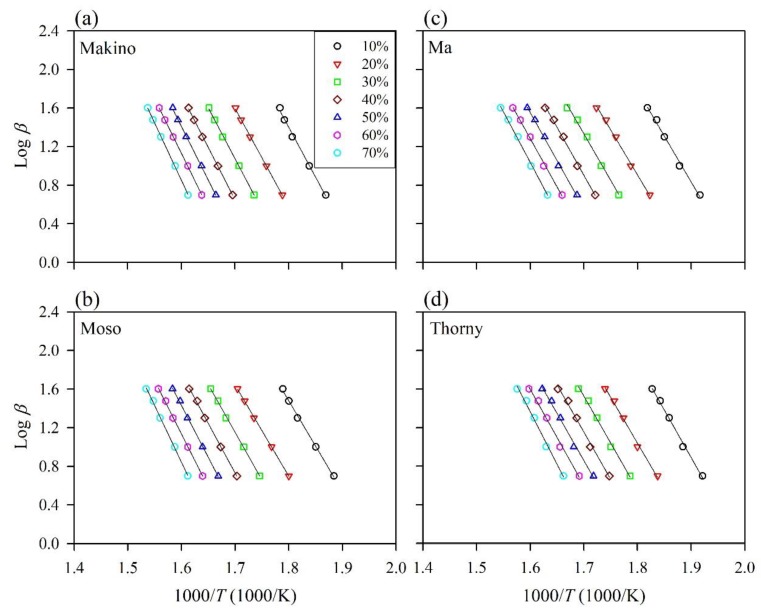
Typical isoconversional plots of various BFs using the Flynn–Wall–Ozawa (FWO) method. (**a**) Makino, (**b**) Moso, (**c**) Ma, and (**d**) Thorny.

To further understand the thermal properties of different BFs, the activation energy (*E*_a_) was determined by the FWO method. Figure 3 shows the plots of the application of the FWO method for conversion rates (*α*) from 10% to 70%. Highly linear fits are remarkably obtained from the plots of log*β* vs. 1/*T* for various BFs, as validated by the square of the correlation coefficient (*R*^2^), which was greater than 0.99 (Table 3). Thus, this method is suitable for determining the *E*_a_ at different *α* for the thermal decomposition processes of various BFs. At a given conversion rate, the *E*_a_ values were calculated from the slope of the linear portion and the intercept of the curve according to Equation (5). According to a previous study [35], the temperature used for composite processing does not exceed 10% conversion. Therefore, the *E*_a_ value at 10% conversion was used to evaluate the thermal stability of the BFs in this study. As shown in Table 3, when the conversion rate reached 10% (*α* = 10%), the temperature was approximately 260 to 290 °C (at heating rates of 5–40 °C/min), which occurred with the decomposition of hemicellulose and the amorphous area of cellulose and the partial decomposition of lignin. At this conversion rate, the *E*_a_ value was approximately 188 kJ/mol for the Makino BF, whereas lower *E*_a_ values (171–173 kJ/mol) were observed for the Moso BF and Ma BF. The higher *E*_a_ value for the Makino BF is related to that this BF contained the lowest extractive content (2.9%). In addition, the lower lignin content (24.5%) for Moso BF and the high quantities of extractives (8.5%) for Ma BF caused lower *E*_a_ values. Accordingly, this result indicated that the Moso BF and the Ma BF were the least thermally stable, while the Makino BF was the most thermally stable. Moreover, these results revealed that the lower lignin content or higher extractive content promoted the thermal decomposition of BFs at relatively low temperatures, reducing the thermal stability of the BF. Furthermore, when *α* = 30–40%, the temperature reached approximately 300–350 °C, corresponding to the decomposition of hemicellulose and cellulose. The Makino BF had an *E*_a_ value close to 200 kJ/mol at conversion rates of 30–40%. However, the *E*_a_ values for the Moso, Ma, and Thorny BFs were nearly 200 kJ/mol up to conversion rates of 60% to 70%. This result confirmed that the Makino BF had the highest thermal stability among all the samples. This result is attributed to the higher CrI value of Makino BF, indicating that the Makino BF contained a higher quantity of ordered cellulose.

**Figure 4:**
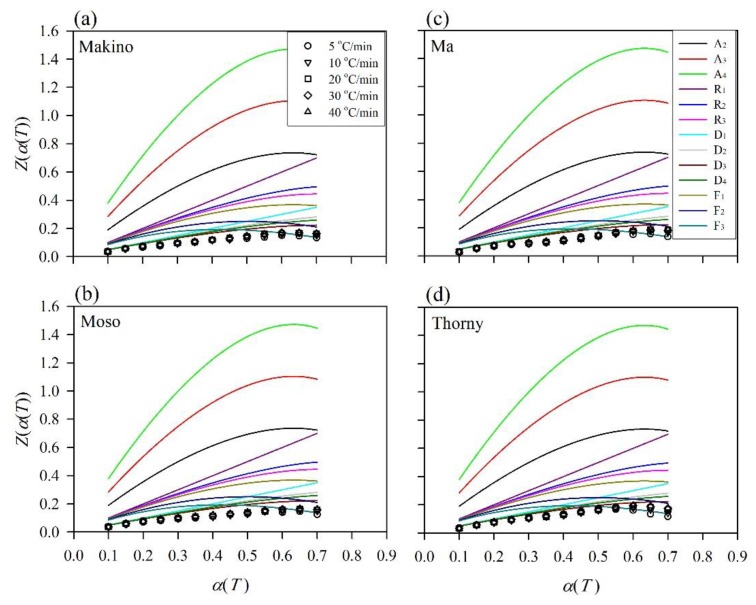
Master curves of kinetic models and experimental data obtained from the Criado method for various BFs. (**a**) Makino, (**b**) Moso, (**c**) Ma, and (**d**) Thorny.

To analyze the thermal decomposition mechanisms of the BFs in depth, the Criado method was used in this study. In this method, the reference theoretical curves, which are called the mater curves (*Z_m_*(*α*)), were obtained from Equation (6). The *Z_m_*(*α*) curves are derivatives of algebraic expressions (*f*(α) and *g*(α)) that represent four groups (A_n_, R_n_, D_n_, and F_n_) of theoretical mechanisms in Table 1. The experimental data, *Z_e_*(*α*(*T*)), for various BFs were calculated by applying the *E*_a_ values obtained from the FWO method (Equation (7)), and these values were determined using a heating rate of 5–40 °C/min. The experimental data and the master curves are compared, and thereby, the mechanism type of the thermally degraded BF can be identified. Figure 4 presents the master curves of the kinetic models and the experimental data for various BFs at conversion rates from 0.1 to 0.7. Comparison of the similarity of these curves yields the kinetic mechanisms throughout the thermal decomposition processes of the different BFs. Furthermore, the experimental data for all of the samples showed the same tendency, regardless of the conversion rate (*α*) reached or the heating rate (*β*) used. At the beginning (0.1 ≤ *α* < 0.3), the plots of the experimental data matched the D_n_ (diffusion-controlled mechanism) curves, which refer to one-, two-, and three-dimensional diffusion. For 0.3 ≤ *α* < 0.5, the *Z_e_*(*α*(*T*)) curves reflected the trend of the D_3_ and D_4_ curves, which corresponds to diffusion in three dimensions (Jander equation and Ginstling–Brounshtein equation). These results implied that the diffusion mechanism becomes prominent for a lower conversion rate (*α* < 0.5). According to previous studies [38,44,45,46], diffusion models are ascribed to the diffusion of gaseous products from thermally degraded samples. Accordingly, the thermal decomposition rates of waste BFs depend on the heat diffusion from the heating source and the diffusion of the formed gases throughout the sample at a lower conversion rate. For higher conversion rates (0.5 ≤ *α* ≤ 0.7), there was a gradual change to the F_3_ mechanism for the experimental data of all the BFs. This mechanism is associated with random nucleation with three nuclei on an individual particle. For these conversion rates, the temperature was higher than 350 °C. Accordingly, the higher temperatures accelerated the cellulose polymer chain into shorter chains. These chains with lower molecular weights could act as sites for random nucleation and growth for degradation reactions. Similar results were reported in the work of Poletto et al. [38] and Singh et al. [46].

### 3.3. Characteristic Properties of the BPCs

**Figure 5:**
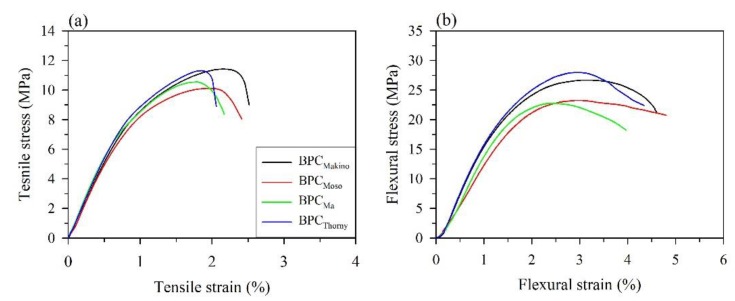
Stress-strain curves obtained from the tensile (**a**) and flexural (**b**) tests for various BPCs.

The density, moisture content (MC), and water absorption rate (WAR) of the BPCs with various BFs are listed in Table 4. Generally, the mechanical properties of materials can be directly influenced by the density. None of the densities of the composites were significantly different (approximately 0.77–0.80 g/cm). In addition, the BPC with Thorny BFs exhibited the highest MC (3.25%) and WAR (10.5%) values. This phenomenon may be affected by the high hemicellulose content in the Thorny BF, which was determined by the TG analysis results. It is well known that hemicellulose is very hydrophilic and has the highest capacity for water absorption, followed by cellulose and lignin [43]. Furthermore, the effects of the various BFs on the mechanical responses of the BPCs are presented in Figure 5 and Table 5. BPC_Makino_ showed the highest tensile strength (TS) and tensile modulus (TM) values at 11.7 MPa and 1130 MPa, respectively, whereas BPC_Moso_ had the lowest tensile properties (10.0 MPa for TS and 987 MPa for TM). For the flexural properties, the moduli of rupture (MORs) of all the samples were approximately 25.0–27.7 MPa; no significant differences were observed among these results. Moreover, the lowest modulus of elasticity (MOE) was found in the BPC with Moso BFs (1482 MPa), followed by BPC_Makino_ (1741 MPa), BPC_Thorny_ (1808 MPa), and BPC_Ma_ (1958 MPa). These results seem to reveal that the mechanical properties of the BPCs were influenced by the intrinsic properties of the BFs such as their structural stiffness and their chemical composition (cellulose and lignin). Jarvis [47] indicated that cellulose has a tight, high strength, and high stiffness crystalline edifice owing to a complex network of hydrogen bonds. The strength and stiffness of natural fibers depend on the crystallinity index, which can represent the cellulose content [20]. Lignin is a branched hydrophobic heteropolymer as a matrix that bonds cellulose fibers together. Additionally, lignin provides strength and stiffness to fiber walls and transfers stress between the cellulose fibers and the matrix [48]. In this study, the Makino BF had a high crystallinity index (53.2%) and lignin content (30.7%), resulting in BPC_Makino_ having the better tensile properties. In contrast, the BPC_Moso_ had the lowest TS, TM, and MOE due to having the lowest lignin content (24.5%) among all the samples. These results illustrated that the Makino BF is a desired reinforcing filler, providing the better tensile properties for a BPC.

## 4. Conclusions

The chemical composition analysis showed that the Makino BF contained the highest holocellulose content and the lowest extractive content, whereas the Moso BF contained the lowest amount of lignin. According to the XRD patterns, the Makino BF had the highest crystallinity index among all of the samples, which means that this BF had the highest cellulose content. Moreover, according to the results of TG analysis, higher hemicellulose and extractive contents promoted the thermal decomposition of the BF at lower temperatures. Moreover, the activation energy (*E*_a_) and kinetic mechanisms for various BFs under the controlled heating of the TG analyses were determined by the FWO method and the Criado method. Among all the BFs used in this study, the Makino BF exhibited the highest *E*_a_ values at various conversion rates, indicating that this BF was the most thermally stable. Using the Criado method, it is noted that the diffusion-controlled mechanism (D_n_ mechanism) was dominant at lower conversion rates (0.1 ≤ *α* < 0.5). When the conversion rate was above 0.5, the degradation of the BFs was governed by the 3rd order random mechanism (a F_3_ mechanism). Furthermore, the BPC with Thorny BFs exhibited the highest moisture content and water absorption rate due to the higher hemicellulose content of the Thorny BF. When a BPC was manufactured with Makino BF, which had high crystallinity and high lignin content, the tensile properties of the composite were high. These results indicated that the amounts of various chemical components within BFs affect the physical and mechanical properties of a BPC. Furthermore, the results of this study offer information for optimizing polymer composites, and the reinforcement of waste BFs needs to be precisely selected in the future.

## Figures and Tables

**Table 1:** Algebraic expressions of the kinetic models for *f*(*α*) and *g*(*α*) for kinetic mechanisms of solid-state processes [28,36].

Kinetic Mechanism	Kinetic Model	Algebraic Expression	
		*f*(*α*)	*g*(*α*)
***Nucleation and growth***			
Avrami equation	A_2_	2(1 − *α*)[ − ln(1 − *α*)]^1/2^	[ − ln(1 − *α*)]^1/2^
Avrami equation	A_3_	3(1 − *α*)[ − ln(1 − *α*)]^2/3^	[ − ln(1 − *α*)]^1/3^
Avrami equation	A_4_	4(1 − *α*)[ − ln(1 − *α*)]^3/4^	[ − ln(1 − *α*)]^1/4^
***Geometrical: Phase boundary-controlled reaction***			
Linear contraction	R_1_	1	*α*
Contracting area	R_2_	2(1 − *α*)^1/2^	1 − (1 − *α*)^1/2^
Contracting volume	R_3_	3(1 − *α*)^2/3^	1 − (1 − *α*)^1/3^
***Diffusion***			
One-dimensional	D_1_	(1/2)*α*	*α* ^2^
Two-dimensional (Valensi equation)	D_2_	[ − ln(1 − *α*)]^−1^	(1 − *α*)ln(1 − *α*)+*α*
Three-dimensional (Jander equation)	D_3_	(3/2)(1 − *α*)^2/3^ [1 − (1 − *α*)^1/3^]^−1^	[1 − (1 − *α*)^1/3^]^2^
Three-dimensional (Ginstling-Brounshtein equation)	D_4_	(3/2)[(1 − *α*)^ − 1/3^ − 1]^−1^	[1 − (2/3)*α*] − (1 − *α*)^2/3^
***Reaction-order: Random nucleation on the individual particle***			
1st order (One nucleus)	F_1_	(1 − *α*)	− ln(1 − *α*)
2nd order (Two nuclei)	F_2_	(1 − *α*)^2^	[(1 − *α*)^−1^] − 1
3rd order (Three nuclei)	F_3_	(1 − *α*)^3^	(1/2)[(1 − *α*)^−2^] − 1

**Table 2:** Chemical compositions of various bamboo fibers (BFs).

Bamboo Species	Chemical Composition		
	Holocellulose (%)	Lignin (%)	Extractives (%)
Makino	62.5 ± 0.8 ^a^	30.7 ± 1.0 ^a^	2.9 ± 0.5 ^d^
Moso	57.1 ± 0.6 ^b,c^	24.5 ± 1.2 ^b^	3.8 ± 0.4 ^c^
Ma	58.0 ± 0.7 ^b^	30.3 ± 0.5 ^a^	8.5 ± 0.5 ^a^
Thorny	56.0 ± 0.6 ^c^	28.1 ± 0.9 ^a^	6.9 ± 0.3 ^b^

Values are the mean ± SD (*n* = 3). Different letters (a, b, c, and d) indicate significant differences (*p* < 0.05).

**Table 3:** Apparent activation energies of various BFs calculated by the FWO method.

Bamboo Species	Items	Conversion Rate (*α*)						
		10%	20%	30%	40%	50%	60%	70%
Makino	*E*_a_ (kJ/mol)	188	186	193	198	202	206	217
	*R* ^2^	0.9960	0.9992	0.9992	0.9995	0.9994	0.9999	0.9997
Moso	*E*_a_ (kJ/mol)	171	172	180	188	196	201	214
	*R* ^2^	0.9979	0.9998	0.9990	0.9988	0.9988	0.9990	0.9986
Ma	*E*_a_ (kJ/mol)	173	170	176	181	181	183	191
	*R* ^2^	0.9941	0.9966	0.9939	0.9969	0.9971	0.9969	0.9969
Thorny	*E*_a_ (kJ/mol)	179	173	177	178	178	182	198
	*R* ^2^	0.9954	0.9948	0.9934	0.9927	0.9937	0.9928	0.9918

**Table 4:** Physical properties of the BPCs with various BFs.

Code	Density (g/cm^3^)	MC (%)	WAR After 24 Soaking (%)
BPC_Makino_	0.78 ± 0.02 ^a^	2.81 ± 0.27 ^b^	6.7 ± 1.7 ^b^
BPC_Moso_	0.80 ± 0.02 ^a^	2.87 ± 0.16 ^b^	5.5 ± 0.3 ^b^
BPC_Ma_	0.77 ± 0.05 ^a^	3.09 ± 0.17 ^a,b^	7.3 ± 1.8 ^b^
BPC_Thorny_	0.78 ± 0.02 ^a^	3.25 ± 0.10 ^a^	10.5 ± 1.6 ^a^

Values are the mean ± SD (*n* = 5). Different letters (a and b) indicate significant differences (*p* < 0.05).

**Table 5:** Mechanical properties of the BPCs with various BFs.

Code	Tensile properties		Flexural properties	
	TS (MPa)	TM (MPa)	MOR (MPa)	MOE (MPa)
BPC_Makino_	11.7 ± 1.0 ^a^	1130 ± 106 ^a^	25.0 ± 2.3 ^a^	1741 ± 156 ^a,b^
BPC_Moso_	10.0 ± 0.4 ^b^	987 ± 68 ^b^	25.1 ± 1.3 ^a^	1482 ± 210 ^b^
BPC_Ma_	10.9 ± 0.7 ^a,b^	1110 ± 85 ^a,b^	27.7 ± 4.5 ^a^	1958 ± 291 ^a^
BPC_Thorny_	11.0 ± 1.0 ^a,b^	1097 ± 65 ^a,b^	26.0 ± 2.1 ^a^	1808 ± 198 ^a^

Values are the mean ± SD (*n* = 8). Different letters (a and b) indicate significant differences (*p* < 0.05).
